# Molecular Dynamic Simulation of the Porcine Pancreatic Lipase in Non-aqueous Organic Solvents

**DOI:** 10.3389/fbioe.2020.00676

**Published:** 2020-07-14

**Authors:** Zi-Shi Chen, Yi-Da Wu, Jin-Heng Hao, Yu-Jia Liu, Kang-Ping He, Wen-Hao Jiang, Mei-Jie Xiong, Yong-Si Lv, Shi-Lin Cao, Jie Zhu

**Affiliations:** ^1^Group of Sustainable Biochemical Engineering, School of Food Science and Engineering, Foshan University, Foshan, China; ^2^School of Chemical Engineering and Energy Technology, Engineering Research Center of Health Food Design and Nutrition Regulation, Dongguan University of Technology, Dongguan, China; ^3^Sustainable Biochemical and Biosynthetic Engineering Center, Foshan Wu-Yuan Biotechnology Co., Ltd., Guangdong Biomedical Industrial Base, Foshan, China

**Keywords:** molecular dynamics simulation, porcine pancreatic lipase, organic solvents, conformation, biocatalysis

## Abstract

This paper investigates the conformational stability of porcine pancreatic lipase (PPL) in three non-aqueous organic solvents, including dimethyl sulfoxide (DMSO), propylene glycol (PRG), and ethanol (EtOH) through molecular dynamic (MD) simulation. The root mean square deviations (RMSDs), radius of gyration (Rg), solution accessible surface area (SASA), radial distribution function (RDF), hydrogen bond (H-bond), Ramachandran plot analysis, secondary structure, and enzyme substrate affinity of the PPL in the various organic solvents were comparatively investigated. The results showed that the backbone and active pocket RMSD, and hydrophilic ASA of PPL in three solvents increase with the increase in the solvent LogP, while the Rg, hydrophobic ASA, and H-bond between the solvent and PPL decrease. Among the three organic solvents, DMSO acts as a better solvent, in which the PPL can be loose and extended, and retains its native backbone in DMSO compared to PRG and EtOH. Moreover, Ramachandran plot analysis indicated that the PPL structure quality in DMSO was higher than that in PRG and EtOH. Also, the molecular docking results showed that PPL in DMSO exhibited the highest enzyme-substrate affinity.

## Introduction

Porcine pancreatic lipase (PPL) is widely used in many industrial fields because of its high enantioselectivity in biocatalysis (Li et al., 2017). This enzyme is composed of 448 amino acid residues, containing a serine-asparate-histidine (Ser153, Asp177, His264) catalytic triad (Hermoso et al., 1996). Comparing with various lipase, PPL is cheaper, which makes it interesting for many applications (Paula et al., 2007).

Organic solvent media provide numerous industrial advantages in lipase-catalytic biosynthesis. According to previous literatures, the choice of organic solvents could strongly affect the catalytic performance of the enzyme (Deng et al., 2016), such as activity, substrate specificity, and enantiopreference (Li et al., 2015). In previous literatures, dimethyl sulfoxide (DMSO) was one of the best solvents for highly efficient and regioselective synthesis of dihydromyricetin esters by immobilized *Penicillium expansum* lipase and Novozyme 435 (Li et al., 2015). Also, literatures reported that DMSO media exhibited potential in reducing protein aggregates and increasing enzyme solubility by avoiding hydrophobic interactions of PPL structure (Vaezzadeh et al., 2018). Thus, elucidating the underlying mechanisms responsible for the observed solvent effects is critical for the bio-catalysis system design. However, the conformational feature of lipase in organic solvents and the mechanism of the effects of solvents on the structure and catalytic performance of the enzyme still await further study.

Molecular dynamic (MD) simulation is a useful pathway in investigating protein structure and obtaining insights into the behavior of the enzymes at the atomic and molecular level. To date, there are several successful examples using MD simulation to illustrate the structure–function relationship of enzymes (Gong et al., 2019). For example, optically pure alcohols are highly valuable chiral chemicals for fine chemical and pharmaceutical industries. Ketoreductase CgKR1 from *Candida glabrata* is one of the greatest potential biocatalysts for large-scale biosynthesis of optically pure alcohols. In order to obtain high-performance CgKR1mutants, Zheng et al. identified two key residues (Phe92 and Phe94) by using MD simulation, molecular mechanics Poisson–Boltzmann (generalized born) surface area method, and molecular docking, and the result indicated that the higher binding affinity between the substrate and enzyme mutants was partially responsible for the improved catalytic enzyme activity. Moreover, combining the computational and experimental results, the authors elucidate a structure–function relationship of mutant CgKR1-F92C/F94W: the indole ring of Trp94 forms a stable π –π stacking interaction with the imidazole ring of His 93, resulting in the significant spatial movement of the substrate (Zheng et al., 2017). Xu et al. investigated the structure–function relationship of the ketoreductase (LbCR) from *Lactobacillus brevis* mutant LbCR-A201D/A202L, and the result indicated that the mutation led to additional hydrogen bonds (H-bonds) formed between the activity pocket and the substrate, resulting in the decrease in *K*_m_ and the activity (Gong et al., 2019). Moreover, the conformations of *Candida antarctica* lipase B in several organic solvents were investigated by molecular simulation previously (Li et al., 2010), but few studies have been focused on PPL.

In this paper, the effect of the three organic solvents, DMSO, propylene glycol (PRG), and ethanol (EtOH), on the conformational change of PPL structure was studied by using MD simulation, in order to give a molecular insight of the PPL structure in the organic solvent.

## Materials and Methods

### General Simulation Approach

The structure of the PPL was obtained from Protein Data Bank (PDB code: 1ETH). The topology formats of DMSO, PRG, and EtOH were generated by the PRODRG for small molecules for the GROMOS96 43A1 force field (Schuttelkopf and Van Aalten, 2004).

The MD simulations were performed with the Gromacs 4.5.3 package (Berendsen et al., 1995) by using the GROMOS 43A1 force field (Schuttelkopf and Van Aalten, 2004). During the MD simulation, the 1ETH PPL was placed into the center of a cubic, in which the PPL is 1 nm apart from the box margin (with the size of 10.81927 nm × 10.81927 nm × 10.81927 nm). Each simulation box contained one 1ETH PPL molecule and given solvent molecules of DMSO, PRG, and EtOH, respectively (Supplementary Table S1). Then, 7 NA+ were added to the box to balance the electrical neutrality of the simulation system. The MD simulation of PPL in the aqueous system is also performed as a control. Firstly, the whole systems were submitted to 50,000 steps of energy minimization at 300 K by using the particle-mesh Ewald method. Then, position-restrained MD simulation was performed to equilibrate the solvent and ions around the protein 1ETH PPL molecule *via* the NVT ensemble (constant Number of particles, Volume, and Temperature), followed by the NPT ensemble (constant Number of particles, Pressure, and Temperature). The PPLs in DMSO, PRG, and EtOH were marked as PPL-DMSO, PPL-PRG, and PPL-EtOH, respectively.

### The Overall Conformation Change of PPL in Organic Solvents

The active pocket of PPL is composed of Gly77, Phe78, Ile79, Asp80, Trp86, Tyr115, His152, Ser153, Leu154, Asp177, Pro181, His264, and Leu265. The active site includes Ser153, Asp177, and His264. The average density of PPL solvents at the last 1 ns was calculated. The average root mean square deviation (RMSD) of the PPL molecules at the last 1 ns was also calculated. The average radius of gyration (Rg) analysis of the PPL molecules backbone at the last 1 ns was also calculated. Secondary structure analysis, including the alpha helix, beta sheet, and the loop content of PPL, was also calculated by PyMol (DeLano, 2002). The hydrophobic, hydrophilic, and total solution accessible surface area (SASA) of PPL at 19- to 20-ns time were calculated. The average H-bond number (HBN) of both PPL solvent pairs at the last 0.1 ns was also calculated. The root mean square fluctuations (RMSFs) of the PPL were averaged for each residue. The Ramachandran plot analysis of the PPL was performed by the PROCHECK software module (Laskowski et al., 1993), deployed in the free online toolbox Magical Platform^1^ constructed by our team. The change of PPL in aqueous as control during molecular simulations. The data outputted from Gromacs were also arranged by using the Magical Platform.

### Molecular Docking and MMPBSA Analysis

The PPL structure (PPL-DMSO, PPL-PRG, and PPL-EtOH) after simulation and the original PPL structure from the PDB database were used as a receptor. Dicaprylin (DAG) was used as the model ligand. AutoDock Vina (Trott and Olson, 2010) was used to dock PPL with DAG ligands, a total of nine models were docking, and the optimal docking results were estimated via the free energy of binding in kcal/mol units. After molecular docking, all of the PPL-DAG composites were analyzed in the protein–ligand interaction profiler (PLIP) module (Salentin et al., 2015). The AutoDock Vina module and the PLIP module were deployed in the free online toolbox Magical Platform^2^ constructed by our team. The molecular mechanics Poisson–Boltzmann surface area (MMPBSA) analysis was performed using the g_mmpbsa tool (Kumari et al., 2014).

### The Overall Conformation Change of PPL in DMSO, SOL, PRG, and EtOH

The partition coefficient of the solvent in between octanol and water can be defined as the oil–water partition coefficient (LogP, polarity constant) of the organic solvent models, which was calculated according to Wang’s method (Cheng et al., 2007). The solvents are defined as non-polar, polar aprotic, and polar protic solvents and ordered by increasing polarity. The results showed that the LogP value of DMSO, PRG, and EtOH were −1.35, −0.92, and −0.09, respectively.

The RMSDs of the PPL molecules in different organic solvents during the simulation process were calculated. As shown in Table 1, for DMSO, PRG, and EtOH solvents, the average RMSD of the PPL molecular backbone at the last 1 ns increased with increasing polarity of the organic solvents (decreasing of the LogP). Relatively speaking, PPL can retain its native backbone in DMSO much better than that in PRG and EtOH.

**TABLE 1:** The overall conformation analysis of PPL in solvents.

Organic Solvent	Density (kg/m^3^)	Backbone RMSD (nm)	Pocket RMSD (nm)	Radius of gyration (nm)	Secondary structure relative content (%)			SASA (nm^2^)		
						
					α -helix	β -sheet	loop	Hydrophobic	Hydrophilic	Total
Water	1,018.3	0.29	0.16	2.47	23.21	27.23	49.55	105.13	90.69	195.82
DMSO	1,083.9	0.21	0.16	2.51	24.55	27.23	48.21	112.72	89.25	201.98
PRG	9,58.2	0.34	0.29	2.45	16.52	22.32	61.16	120.47	63.1	183.57
EtOH	707.5	0.43	0.35	2.34	16.07	21.43	62.50	128.89	56	184.89

Table 1 also indicated that the Rg of the PPL decreased with decreasing polarity of solvents. The Rg of PPL-SOL and PPL-DMSO were 2.47 and 2.51 nm, while that of PPL-PRG and PPL-EtOH were 2.45 and 2.34 nm, respectively. Rg is considered to be an indicator of protein structure compactness (Tarazona et al., 1992). The compactness of a protein depends on the hydrophobicity of its chain (Kohn et al., 2004), while the hydrophobicity of a protein chain was significantly influenced by the polarity of the surrounding solvent (Brady et al., 1990). A previous study showed that the protein could be highly compressed by solvent pressure in the poor solvent, resulting in a solid-like appearance. As a comparison, in a good solvent, the protein is loose and extended (Kohn et al., 2004).

According to the surface area analysis result of the PPL molecules, the hydrophobic surface area of the PPL in the three organic solvents was bigger than that in the aqueous solution (104.84 nm^2^). It is worth noting that the hydrophilic surface area of the PPL in the organic solvent increased gradually as the polarity of the organic solvents decreased. Also, compared to PPL in the aqueous solution, the ratio of the hydrophobic area to the total surface area increases significantly, revealing the hydrophobic part of the protein molecules and thus becomes more accessible. Thus, combining the Rg analysis of the PPL in the presence of different organic solvents, these results demonstrate that the compression of the PPL molecules (the decreasing of the Rg) in the organic solvents was mainly due to the exposure of the hydrophobic part of the protein on their surface.

**FIGURE 1:**
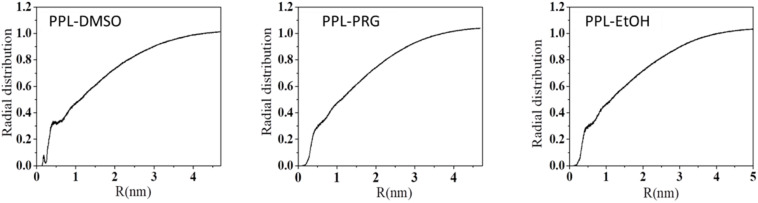
Radial distribution function (RDF) of solvent molecule to PPL.

Overall, the presence of organic solvent molecules around the enzyme can be interpreted from the radial distribution function (RDF) of solvent molecule to PPL. The first RDF peak at about 0.3 nm belongs to the H-bond between the amino acid residues of the PPL and the solvents (Figure 1).

The total number of the H-bond between PPL and the solvent is shown in Supplementary Figure S7. The results showed that the total number of H-bond between the solvent and enzyme decreased with the increase in the organic solvent’s hydrophobicity. The HBN between water and PPL was about 762. For the three organic solvents, the H-bond between PPL of DMSO was about 211, while that of PRG and EtOH was only 66 and 40, respectively. In order to throw insights into these H-bond results, the number of the DMSO, PRG, and EtOH surrounding the PPL was analyzed, with the number 497, 392, and 362, respectively. Moreover, combining the SASA and Rg data of DMSO, PRG, and EtOH during the simulation, the highest number of H-bond between DMSO and PPL can be explained as follows: among the three organic solvents, the PPL in the DMSO solvent exhibits the highest SASA and Rg. Thus, the PPL can contact with more solvent molecules, resulting in more possibility of forming H-bonds.

Based on the secondary structure analysis, the alpha helix structure of the PPL decreased with the increase in the polarity of the organic solvent. Moreover, the beta sheet structure content of the PPL in DMSO was similar with the PPL water and significantly higher than that in PRG and EtOH. What’s more, the loop content in the three organic solvents increased from 48.21 to 62.50%, with the increase in the solvent LogP.

**FIGURE 2:**
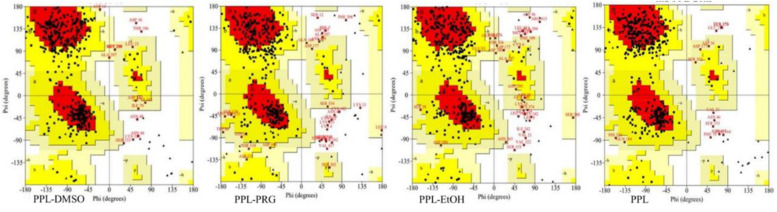
Ramachandran plot analysis of PPL in different solvents.

Ramachandran plot is an objective method of evaluating the quality of a protein structure, as shown in Figure 2 and Supplementary Table S1. The results indicated that the number of amino acids of PPL in DMSO (276) located at the most favored region was significantly higher than that in PRG (236) and EtOH (225), while the amino acid located at the disallowed regions was significantly lower than that in PRG and EtOH. This indicates that the quality of the PPL structure in DMSO was higher than that in PRG and EtOH.

**FIGURE 3:**
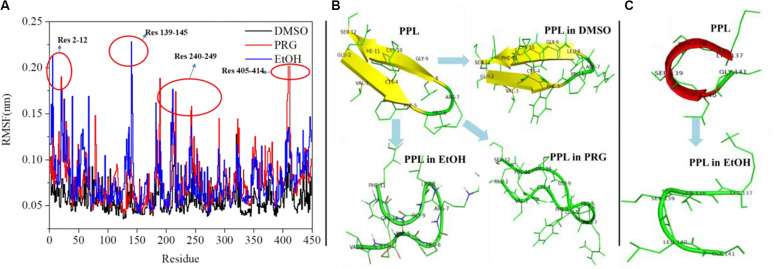
**(A)** RMSF analysis of PPL in organic solvents; **(B)** structure analysis of region (Glu2-Ser12); **(C)** structure analysis of region (Lys137-Gly141).

Figure 3A reveals the RMSF of the PPL in DMSO, PRG, and EtOH. For PPL in all three organic solvents, the regions (Lys70-Val125), (Ile150-Glu180), and (Gly255-Pro285) exhibited a relatively low RMSF value, indicating lower flexibility and higher rigidity of these regions. It is worthy to note that the active pocket of PPL (includes Gly77, Phe78, Ile79, Asp80, Trp86, Tyr115, His152, Ser153, Leu154, Asp177, Pro181, His264, and Leu265) is located at the above regions. Thus, the overall conformation of the active pocket remains stable during the simulation.

Moreover, for PPL in PRG and EtOH, the regions (Glu2-Ser12), (Lys137-Gly141), (Cys182-Val190), (Lys240-Ile249), (Asn320-Val325), and (Asn405-Val414) exhibited a relatively high RMSF value than that of PPL in DMSO, indicating higher flexibility and lower rigidity of these regions. The region (Glu2-Ser12) of PPL-DMSO belongs to a beta sheet, while that of PPL-PRG and PPL-EtOH belongs to a loop structure (Figure 3B). On the other hand, the significant RMSF peak that occurred at (Lys137-Gly141) in PPL-EtOH mainly attributed to the conversion from the alpha helix to the loop structure while in the presence of the EtOH solvent (Figure 3C). The regions (Lys240-Ile249) and (Asn320-Val325), which mainly consist of the loop structure, also exhibit RMSF peaks.

### Effect of the Solvent on the Enzyme-Substrate Affinity

Molecular docking is a useful way of investigating enzyme substrate affinity. As shown in Supplementary Table S1, the PPL-DMSO exhibits the highest affinity (−6.1 kcal/mol) among the three organic solvents, suggesting that PPL-DMSO has the most stable docked structure compared to that of the other solvents.

Moreover, PLIP was used to investigate the PPL–substrate interaction. The greatest number of the substrate-binding sites can be observed in PPL-DMSO. The number of amino acid residues demonstrating hydrophobic interactions with the DAG substrate in PPL-DMSO was six, while that in PPL-PRG and PPL-EtOH were both five. Three residues in PPL-DMSO were detected to interact with DAG through hydrogen bonding, while in PPL-PRG and PPL-EtOH, there was only one. What’s more, two residues in PPL-DMSO can interact with DAG *via* the salt bridge, while none of the salt bridge can be observed between DAG and PPL-PRG/PPL-EtOH. These results illustrated that the PPL in the DMSO solvent exhibited the highest enzyme-substrate affinity compared to PRG and EtOH.

After simulating 20 ns under the same conditions, the free energy results of the three organic solvents were calculated by the MM/PBSA method (Supplementary Table S5). The total binding energy results of the three PPL–ligand complexes were negative, indicating that there was a spontaneous adsorption process between PPL and ligand LIG in the three organic solvent models. Moreover, among the three organic solvents, the highest total binding affinity was observed in the DMSO-PPL model (−115.737 ± 14.157 kJ/mol), followed by the ETO organic solvent model (−105.076 ± 27.058 kJ/mol), and the lowest was the ETO organic solvent model (−72.215 ± 49.967 kJ/mol). Comparing with the different interaction energy (including van der Waals energy, electrostatic energy, polar solvation energy, and SASA energy), the van der Waals interactions are dominant in the PPL-LIG binding process. On the contrary, polar solvation has a strong antagonistic effect on the combination of PPL and LIG.

## Conclusion

In this paper, the conformation, structure, and substrate affinity in the three organic solvents are discussed. *Via* simulation, the RMSDs, Rg, SASA, RDF, H-bond, Ramachandran plot analysis, secondary structure, and the enzyme substrate affinity of the PPL in the various organic solvents were comparatively investigated. The results showed that the backbone, active pocket RMSD, and hydrophilic ASA of PPL in the three solvents increase with the increase in the solvent LogP, while the Rg, hydrophobic ASA, and H-bond between the solvent and PPL decrease. Among the three organic solvents, DMSO was the better solution compared to PRG and EtOH for the following reasons: (1) PPL can be loose and extended and retained its native backbone in DMSO, (2) higher PPL structure quality as indicated by Ramachandran plot analysis, and (3) PPL in the DMSO solvent exhibited the highest enzyme-substrate affinity according to molecular docking results and MMPBSA analysis.

## Data Availability Statement

The raw data supporting the conclusions of this article will be made available by the authors, without undue reservation, to any qualified researcher.

## Author Contributions

Z-SC, Y-DW, and S-LC conceived and designed the experiments. Z-SC, Y-DW, and K-PH performed the experiments. Y-DW, Y-JL, W-HJ, M-JX, and Y-SL analyzed the data. Z-SC, Y-DW, JZ, and S-LC wrote the manuscript. All authors contributed to the article and approved the submitted version.

## Conflict of Interest

S-LC was part-time employed by the company Foshan Wu-Yuan Biotechnology Co., Ltd. The remaining authors declare that the research was conducted in the absence of any commercial or financial relationships that could be construed as a potential conflict of interest.

## Footnotes

1 https://mag.liulianpisa.top/

2 https://mag.liulianpisa.top/

## Supplementary Material

FIGURE S1 PLIP analysis of PPL–DAG interaction.

FIGURE S2 Backbone RMSD of PPL in different solvents during the simulation.

FIGURE S3 Pocket RMSD of PPL in different solvents during the simulation.

FIGURE S4 Hydrophobic SASA of PPL in different solvents during the simulation.

FIGURE S5 Hydrophilic SASA of PPL in different solvents during the simulation.

FIGURE S6 Total SASA of PPL in different solvents during the simulation.

FIGURE S7 H-bond analysis of PPL between different solvents during the simulation.

TABLE S1 Quantitative analysis of different solvent models.

TABLE S2 Ramachandran plot analysis of PPL in different solvents.

TABLE S3 Molecular docking analysis performed by AutoDock Vina.

TABLE S4 Enzyme–substrate interaction analysis by PLIP.

TABLE S5 G_mmpbsa analysis of the PPL–DAG complex in different solvents.
