# Supplementary material for: Molecular Dynamic Simulation of the Porcine Pancreatic Lipase in Non-aqueous Organic Solvents
Source: Front Bioeng Biotechnol. 2020 Jul 14;8:676. doi: 10.3389/fbioe.2020.00676 (PMC7381131; doi:10.3389/fbioe.2020.00676)
Supplement: FIGURE S1 — PLIP analysis of PPL–DAG interaction. [file Data_Sheet_1.pdf]

Supplementary Material

1 Supplementary Figures and Tables

1.1 Supplementary Figures

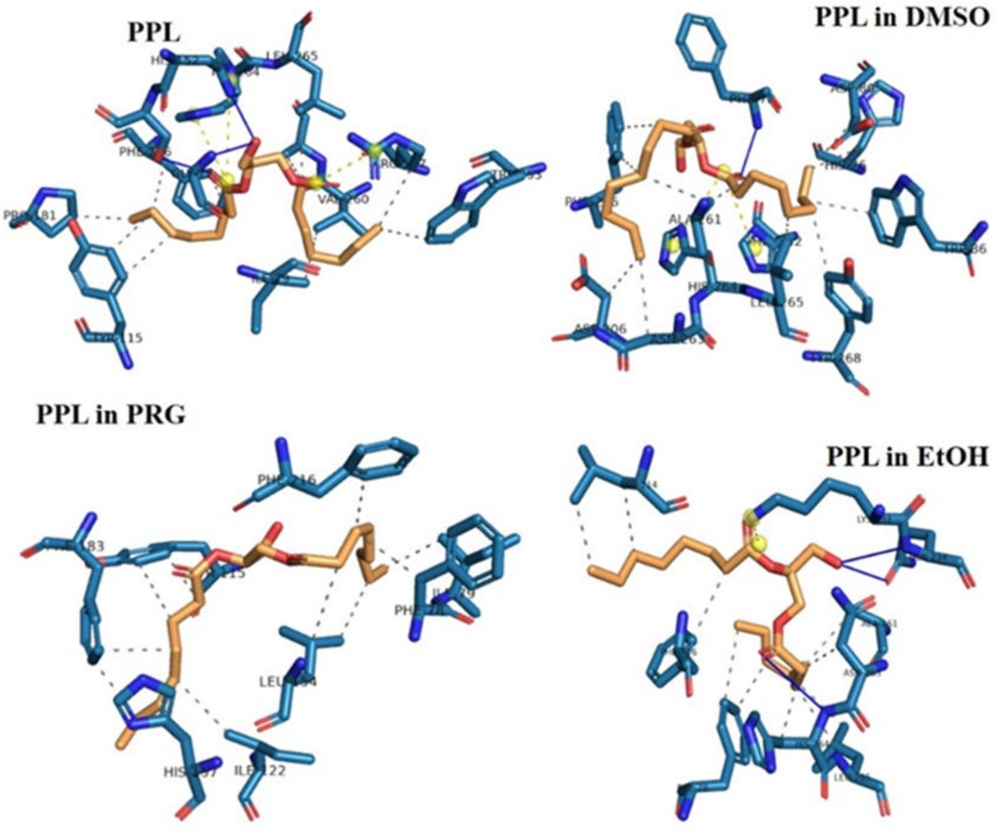

Figure S1 PLIP analysis of PPL-DAG interaction.

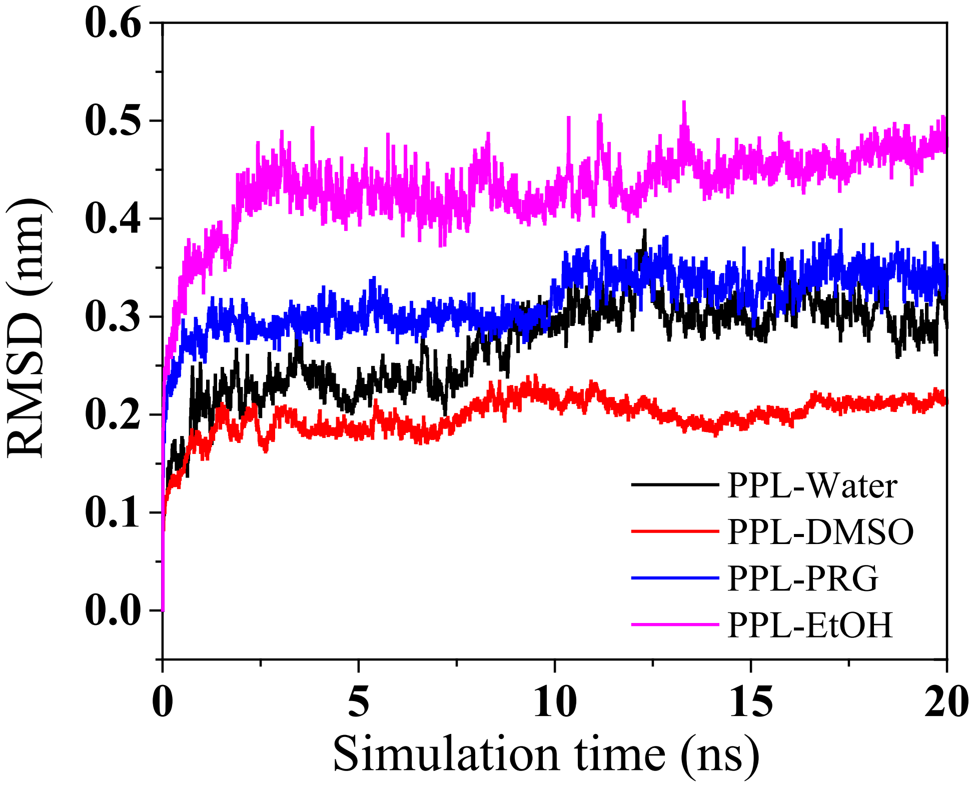

Figure S2 Backbone RMSD of PPL in different solvents during the simulation.

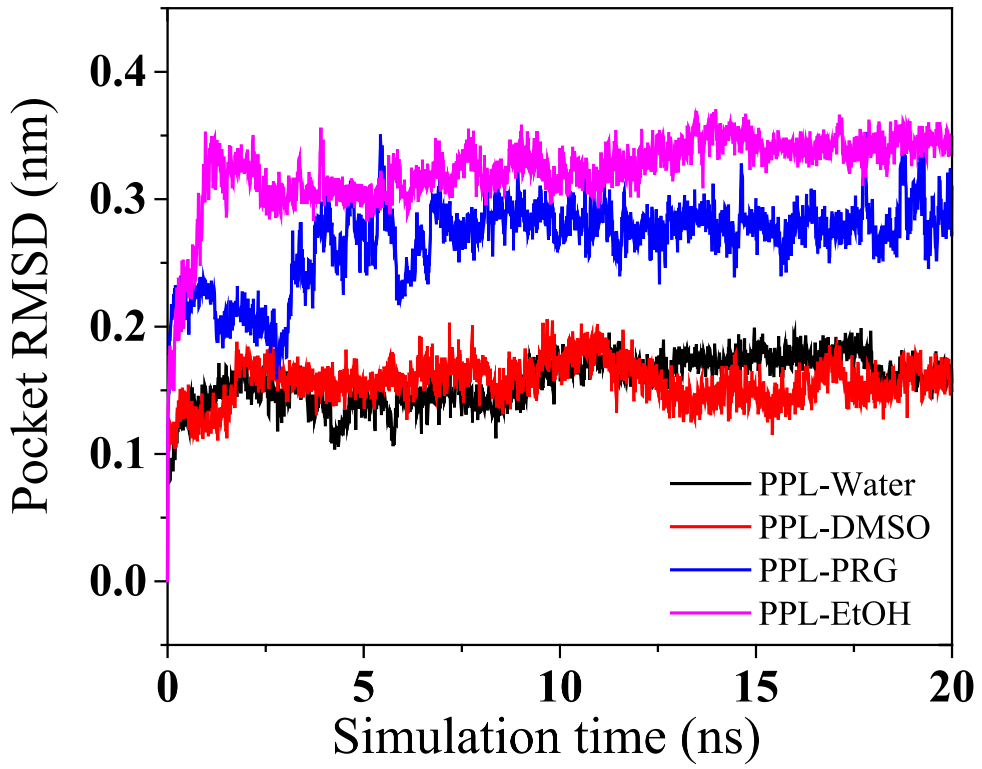

Figure S3 Pocket RMSD of PPL in different solvents during the simulation.

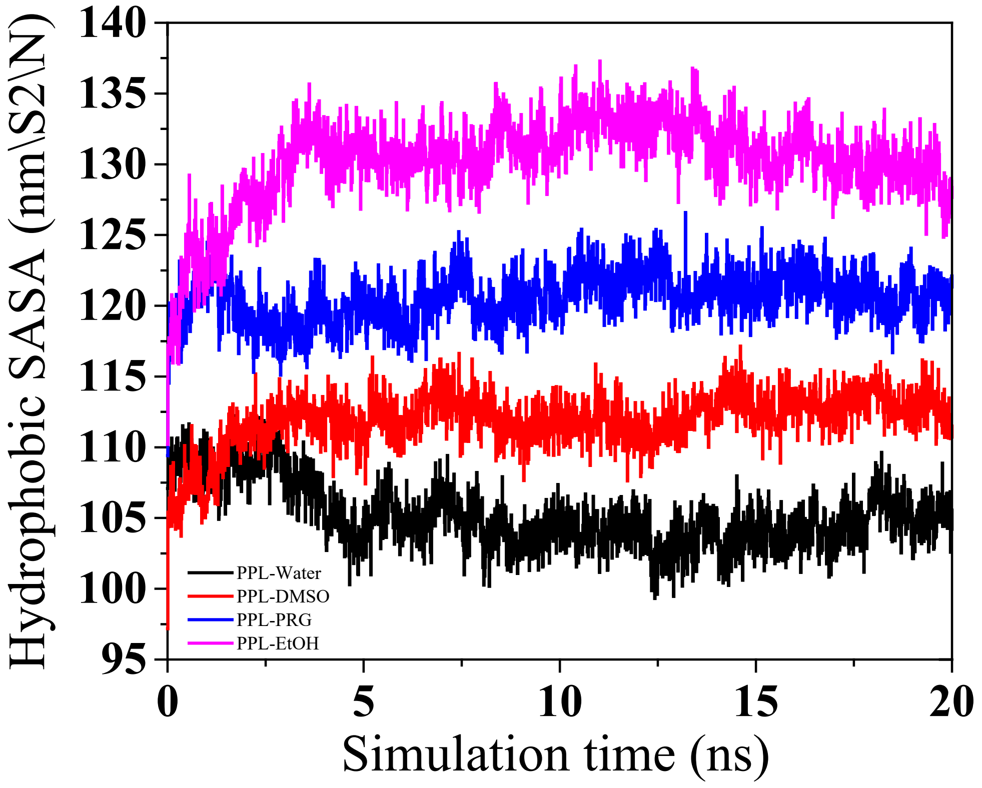

Figure S4 Hydrophobic SASA of PPL in different solvents during the simulation.

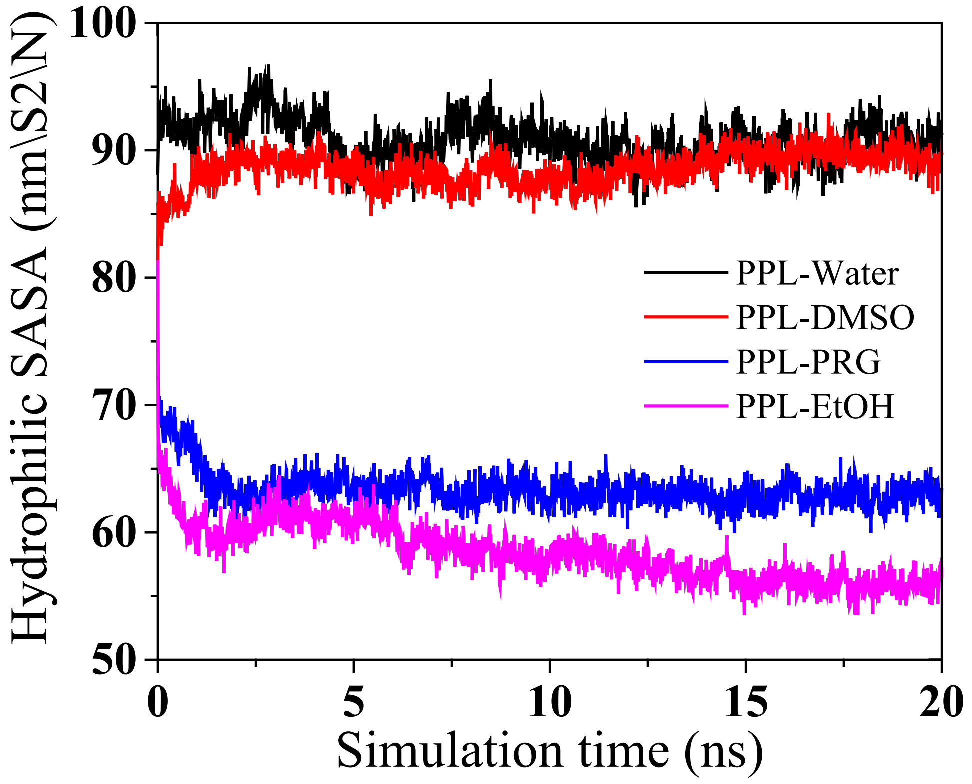

Figure S5 Hydrophilic SASA of PPL in different solvents during the simulation.

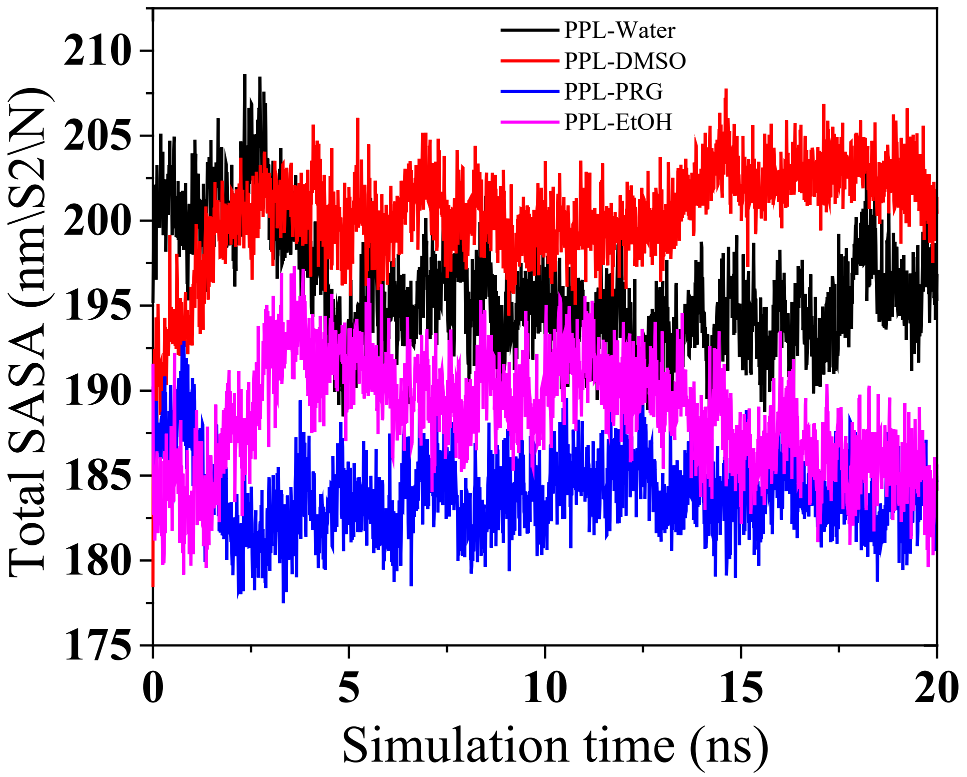

Figure S6 Total SASA of PPL in different solvents during the simulation.

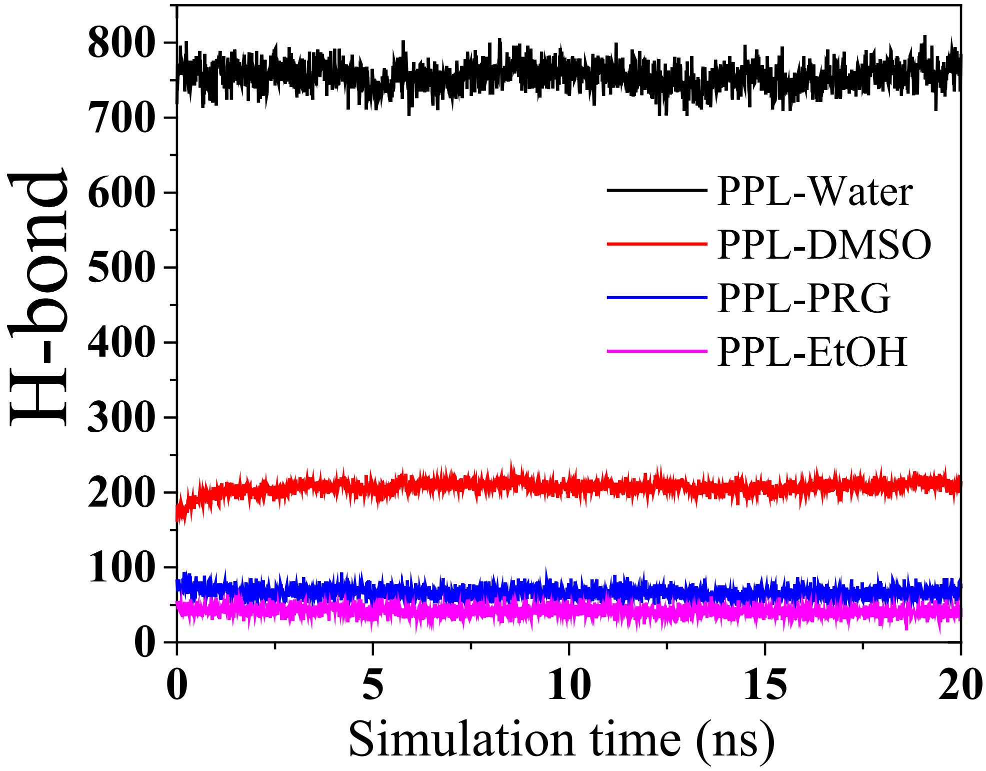

Figure S7 H-bond analysis of PPL between different solvents during the simulation.

1.2 Supplementary Tables

Table S1 Quantitative analysis of different solvents models.

System	Protein (number)	Solvents category	Solvents (number)	Ions-NA$^+$ (number)
PPL	1	Water	39977	7
PPL-DMSO	1	DMSO	10326	7
PPL-PRG	1	PRG	5767	7
PPL-EtOH	1	EtOH	9341	7

Table S2 Ramachandran Plot Analysis of PPL in different solvents.

Regions	PPL	PPL-DMSO	PPL-PRG	PPL-EtOH
Most Favoured	328	276	236	225
Additional Allowed	47	92	114	125
Generously Allowed	5	7	15	17
Disallowed	2	7	17	15

Table S3 Molecular docking analysis performed by Autodock Vina.

PPL structure	Ligands	LogP	Affinity (kcal/mol)
PPL	DAG	/	-6.4
PPL-DMSO		-1.39	-6.1
PPL-PRG		-0.92	-6.0
PPL-EtOH		-0.09	-4.6

Table S4 Enzyme- Substrate Interaction analysis by PLIP

	Hydrophobic Interactions	Hydrogen Bond
PPL_crystal	Ile79, Tyr115, Pro181, Phe216, Trp253, Arg 257, Val260, Ala261, Leu265	Gly77, His152, Ser153
PPL-DMSO	His76, Asp80, Trp86, Asp206, Phe216, Ala261, Asn263, Leu265, Tyr268	Phe78
PPL-PRG	Phe78, Ile79, Tyr115, Ile122, Leu154, Phe183, Phe216	/
PPL-EtOH	Phe78, Leu214, Phe216, Ala261, Asn263, His264, Leu265,	Gln234, His264

Table S5 G_mmpbsa analysis of PPL-DAG complex in different solvents.

PPL structure	van der Waal energy （KJ/mol）	Electrostattic energy （KJ/mol）	Polar solvation energy （KJ/mol）	SASA energy （KJ/mol）	Binding energy （KJ/mol）
Water	-185.326+/-38.107	-15.526+/-7.797	76.232+/-35.079	-17.545+/-3.622	-142.165+/-64.834
DMSO	-134.387+/-15.792	-18.823+/-11.964	51.148+/-12.637	-13.676+/-1.289	-115.737+/-14.157
ETO	-125.815+/-15.003	-40.933+/-7.716	72.303+/-23.079	-10.631+/-1.903	-105.076+/-27.058
PRG	-84.35+/-53.533	-13.832+/-9.818	34.529+/-21.599	-8.563+/-4.576	-72.215+/-49.967
